# Circulation of thermophilic *Campylobacter* in pigeons, turkeys, and humans at live bird markets in Egypt

**DOI:** 10.3389/fvets.2023.1150077

**Published:** 2023-05-12

**Authors:** Amal S. M. Sayed, Ahmed I. Ibrahim, Mona M. Sobhy, Ehab Kotb Elmahallawy, Noorah Alsowayeh, Khaloud Mohammed Alarjani, Manal F. El-khadragy, Asmaa Gahlan Youseef

**Affiliations:** ^1^Department of Zoonoses, Faculty of Veterinary Medicine, Assiut University, Asyut, Egypt; ^2^Poultry Diseases Department, Faculty of Veterinary Medicine, South Valley University, Qena, Egypt; ^3^Reproductive Diseases Department, Animal Reproduction Research Institute, Giza, Egypt; ^4^Department of Zoonoses, Faculty of Veterinary Medicine, Sohag University, Sohag, Egypt; ^5^Department of Biology, College of Education (Majmaah), Majmaah University, Al Majma’ah, Saudi Arabia; ^6^Department of Botany and Microbiology, College of Science, King Saud University, Riyadh, Saudi Arabia; ^7^Department of Biology, College of Science, Princess Nourah bint Abdulrahman University, Riyadh, Saudi Arabia; ^8^Zoonoses Department, Faculty of Veterinary Medicine, South Valley University, Qena, Egypt

**Keywords:** *Campylobacter*, pigeon, Turkey, human, live bird market, Egypt

## Abstract

Live bird markets increase the risk of transmission of zoonotic diseases. Few studies have investigated the potential zoonotic transmission of *Campylobacter* in Egypt. Therefore, our study was carried out to investigate the presence of *Campylobacter* species, mainly *Campylobacter jejuni* (*C. jejuni*) and *Campylobacter coli* (*C. coli*), in pigeons and turkeys sold at poultry shops. Furthermore, the study aimed to explore the potential occupational risk of *Campylobacter* infection, mainly among workers at poultry shops. Six hundred (*n* = 600) samples from various organs were obtained from pigeons and turkeys from live bird shops in the Giza and Asyut provinces in Egypt. Additionally, 100 stool samples were collected from persons working at poultry shops. Circulation of thermophilic *Campylobacter* in pigeons, turkeys, and humans was investigated based on culture and molecular methods. The rate of detection of *Campylobacter* species from the samples was significant when the culture method was used alone in comparison to when it was used in combination with mPCR. The prevalence rates of *Campylobacter* species detected by mPCR were 36% (*C. jejuni* 20%; *C. coli* 16%), 28% (*C. jejuni* 12%; *C. coli*16%), and 29% (*C. jejuni* 15%; *C. coli* 14%) in pigeons, turkeys, and workers, respectively. In pigeons, significant variations in the *C. jejuni* and *C. coli* occurrence rates were reported in terms of the intestinal content (15, 4%), liver (4, 13%), and skin (9, 7%), respectively. In turkeys, *Campylobacter* species were mostly detected in liver samples with a percentage of 19%, followed by the skin (12%), and the intestinal content (8%). In conclusion, *Campylobacter* species are circulating in poultry farms in Egypt and could represent a hazard for humans. It is recommended that biosecurity measures should be applied to mitigate the occurrence of *Campylobacter* in poultry farms. Moreover, there is an urgent need to transform live bird markets into chilled poultry markets.

## 1. Introduction

Foodborne gastroenteritis, caused by *Campylobacter* is a bacterial diarrheal disease that is found worldwide (1). In developing countries, the *Campylobacter* infection rate varies from 5 to 20% (2). Animals and poultry are implicated in zoonotic transmission to humans (3). The bird intestine is considered the best habitat for the multiplication of *Campylobacter* species (4). Cross-contamination of poultry meat with *Campylobacter* usually occurs during evisceration (5). The handling of raw poultry and the consumption of undercooked poultry meat are the main sources of *Campylobacter* infection in humans (6). In relation to its clinical picture, *Campylobacter* infection in humans is usually mild and reported as sporadic cases (7–9). However, community outbreaks of *Campylobacter* have also been reported, and some patients may develop severe illness (10). In addition, *C. jejuni* infection is associated with Guillain–Barre syndrome and reactive arthritis (11).

Several studies reported the presence of *Campylobacter* in the intestine of asymptomatic persons in developing countries (12, 13). Among others, *C. jejuni* is the major cause of *Campylobacter* gastroenteritis in humans, followed by *C. coli*, and to a lesser extent, *Campylobacter lari* (14). In Egypt, the current incidence of *Campylobacter* enteritis in humans is still unclear due to a lack of national surveillance programs, so the majority of reported human cases have come from research studies. Several investigators have reported variable incidence rates of 2.3 and 9.6% for *Campylobacter* (15, 16). It is noteworthy to mention that 90% of the chicken meat in Egypt is produced by commercial poultry farms. However, 10% of the meat is provided by small breeders. Additionally, ducks, geese, pigeons, and turkeys are produced in the backyards of villagers which are produced either mainly for self-consumption or sell (17). Moreover, the live poultry trade is considered the principal strategy for the retail of poultry and covers about 60–80% of the overall poultry commercial production. Live birds are sold commercially in poultry shops distributed all over the country (17). Live bird markets (LBMs) increase the risk for the transmission of zoonotic diseases to humans. To our knowledge, very limited information is available on the occurrence of thermophilic *Campylobacter* in pigeons, turkeys, and humans at live bird markets in Egypt. Therefore, our study was designed to investigate the presence of *Campylobacter* in pigeons and turkeys sold in poultry shops. In addition, we investigated persons working at poultry shops to elucidate the occupational risk of *Campylobacter* infection.

## 2. Materials and methods

### 2.1. Ethical statement

The present study was approved by Assiut University, Egypt under the approval number 1717300906.

### 2.2. Study area and sample collection

Six hundred samples were collected from pigeons and turkeys (100 each) from live poultry markets in the Giza and Asyut provinces in Egypt in the period from August 2014 to December 2019. Three samples (intestinal content, liver, and skin) were obtained from each bird. In addition, 100 stool samples were examined from healthy persons working at poultry shops. The workers were aged 18–50 years.

### 2.3. Isolation and biochemical identification of *Campylobacter*

In this step, *Campylobacter* species were isolated in accordance with ISO 10272-2 (18). Samples from the skin or liver, or 10 g of the intestinal content or stools were homogenized in Bolton broth. Samples were then enriched in Bolton broth (Oxoid) and incubated at 37°C for 4–6 h. Then, they were kept at 41.5°C in a microaerophilic atmosphere for 48 h (Campygen; Oxoid). A loopful of the enrichment broth was plated in modified charcoal cefoperazone *Campylobacter* desoxycholate agar (mCCDA) (Oxoid) and incubated at 41.5°C under microaerobic conditions for 48 h. Colonies were identified according to the procedure mentioned in ISO10272-2 (18).

### 2.4. Molecular identification of *Campylobacter jejuni* and *Campylobacter coli*

#### 2.4.1. Extraction of DNA

The DNA of each strain was extracted using the Genomic DNA Purification Kit (Thermo Scientific Gene Jet Purification Kit#K0721, #K0722) in accordance with the manufacturer’s instructions. Briefly, a purified colony was inoculated into tryptic soya broth and incubated at 37°C for 48 h. Bacterial cells (2 × 10^9^) were harvested in a 1.5 ml microcentrifuge tube by centrifugation at 5000 × g for 10 min. The pellet was suspended in a solution composed of 180 μl of digestion solution and 20 μl of proteinase K, which was then mixed thoroughly at 56°C. RNase A (20 μl) was added. The solution was mixed and then incubated at room temperature for 10 min. Lysis solution (200 μl) was added and the solution was mixed thoroughly by vortexing for about 15 s. Then, 400 μl of ethanol (50%) was added. The solution was mixed and then transferred to a purification column with a collection tube and centrifuged for 1 min at 6000 × g. Collection tubes were then discarded and replaced by 2 ml collection tubes. Washing buffer I (500 μl) was then added, and the solution was centrifuged for 1 min at 8000 × *g*. Wash buffer II (500 μl) was added, and the solution was centrifuged for 3 min at maximum speed (≥12,000 × *g*). The elution buffer (200 μl) was added to the center of the purification column membrane, and the solution was incubated for 2 min at room temperature and centrifuged for 1 min at 8000 × *g*.

#### 2.4.2. Polymerase chain reaction

This step involved the multiplex polymerase chain reaction (mPCR). Three primers targeting the *Campylobacter 23S rRNA* gene, the *hip O* gene for *C. jejuni*, and the *glyA* gene for *C. coli* were used to amplify 650, 323, and 126 bp (Supplementary Figure S1), respectively, as described previously (19). PCR was carried out in a volume of 50 μl with 25 μl of the master mix, 10 μl of the DNA template (50 ng), 9 μl of grade water, and 1 μl of each primer (20 pmol). The PCR conditions were as described previously (19), as follows: there was an initial denaturation step of 94°C for 6 min, followed by 35 cycles, each consisting of 30 s at 95°C, 30 s at 59°C, 30 s at 72°C, and a final extension step at 72°C for 7 min. The electrophoresis of PCR products was performed in 1.5% agarose at 80 V in Tris base–boric acid–EDTA buffer for 120 min. The UV trans-illuminator (Biometra) was used for the visualization of amplicons. Then, they were photographed with the Gel Documentation System using BioDocAnalyze software. A negative control and a positive control were also included.

### 2.5. Statistical analysis

SAS version 9.4 was used to analyze the data. The Chi-square test was used identify the level of significance, and *p-*values of <0.05 were considered significant.

## 3. Results

### 3.1. *Campylobacter* species in pigeons, turkeys, and humans

Table 1 shows that the overall prevalence of *Campylobacter* species determined by the culture method alone and in combination with mPCR was 36.3% (109/300) and 31% (93/300), respectively. The prevalence of *C. jejuni* determined by the culture method alone and in combination with mPCR was 26.7% (80/300) and 15.7% (47/300), respectively, and the difference was statistically significant (*p* = 0.001). In addition, as depicted in Table 1, the prevalence of *C. coli* determined by the culture method alone and in combination with mPCR was 9.7% (29/300) and 14.7% (44/300), respectively, and the difference was statistically significant (*p* = 0.0359). The prevalence rates of *Campylobacter* species detected by mPCR were 36% (*C. jejuni* 20%; *C. coli* 16%), 28% (*C. jejuni* 12%; *C. coli* 16%), and 29% (*C. jejuni* 15%; *C. coli* 14%) in pigeons, turkeys, and humans, respectively, and the differences were not statistically significant.

**Table 1:** Identification of *Campylobacter* species using the culture method and PCR.

Source of samples	Number of samples	Culture method						Culture and PCR					
		*Campylobacter* species		*C. jejuni*		*C. coli*		*Campylobacter* species		*C. jejuni*		*C. coli*	
		No.	%	No	%	No.	%	No.	%	No.	%	No.	%
Pigeons	100	42	42	28	28	14	14	36	36	20	20	16	16
Turkeys	100	35	35	24	24	11	11	28	28	12	12	16	16
Poultry shop workers	100	32	32	28	28	4	4	29	29	15	15	14	14
Total	300	109	36.3	80	26.7^a^	29	9.7^b^	93	31	47	15.7^a^	44	14.7^b^

^a^*p* = 0.001. ^b^*p* = 0.0359.

### 3.2. Distribution pattern of *Campylobacter* species isolates in pigeons

As shown in Table 1, *Campylobacter* species were detected in 17.3% (52/300) of the examined pigeons, of which 19, 17, and 16% of the positive samples were from the liver, intestinal content, and skin, respectively. *C. jejuni* was detected in the intestinal content (15%), liver (4%), and skin (9%) samples, and the differences between sample types were statistically significant (*p* = 0.0277). *C. coli* was identified in the intestinal content (4%), liver (13%), and skin (7%) samples, and the differences between sample types were statistically significant (*p* = 0.0577) (Table 2). Twenty-eight isolates of *C. jejuni* were isolated from 20 pigeons (Tables 1, 2), and different patterns were shown. Two isolates of *C. jejuni* were retrieved from both the liver and the skin samples of four pigeons (Nos. 6, 7, 15, 16), as shown in Table 3. However, separate isolates of *C. jejuni* were isolated from the intestine or skin samples of the remaining pigeons (Table 3). Furthermore, twenty-three isolates of *C. coli* were isolated from 16 pigeons (Tables 1, 2), and different patterns were shown. *C. coli* was isolated from the intestinal content, liver, and skin samples of the same pigeon (No.1), and *C. coli* was isolated from both the intestinal content and the skin samples of six pigeons (Nos. 5, 10, 11, 12, 13, and 14). Separate isolates were isolated from the intestine and skin samples of the remaining pigeons (Table 4).

**Table 2:** Distribution pattern of *Campylobacter* species isolates in pigeons and Turkeys.

Type of samples	Number of samples	*Campylobacter* species		*Campylobacter jejuni*		*p-value*	*Campylobacter coli*		*P-value*
		No.	%	No.	%	0.0277	No.	%	0.0577
(A) Pigeon									
Intestinal content	100	19	19	15	15		4	4	
Liver	100	17	17	4		4	13	13	
Skin	100	16	16	9		9	7	7	
Total	300	52	17.3	28	9.33		23	7.7	
(B) Turkey									
Intestinal content	100	8	8	4	4	*p* > 0.05	4	4	*p* > 0.05
Liver	100	19	19	8	8		11	11	
Skin	100	12	12	4	4		8	8	
Total	300	39	13	16	41.03		23	7.7	

**Table 3:** Distribution pattern of *C. jejuni* isolates in pigeons and Turkey.

Bird and its serial number	Source of samples			Bird serial number	Source of samples		
	Intestinal content	Liver	Skin		Intestinal content	Liver	Skin
(A) Pigeon							
1	+	−	−	11	−	−	+
2	+	−	−	12	+	−	+
3	+	−	−	13	+	−	+
4	−	−	+	14	−	−	+
5	−	−	+	15	−	+	+
6	−	+	+	16	−	+	+
7	−	+	+	17	+	−	+
8	+	−	−	18	+	−	+
9	+	−	−	19	+	−	−
10	−	−	+	20	+	−	−
(B) Turkey							
1	−	−	−	7	+	−	−
2	+	+	+	8	−	−	−
3	+	−	−	9	+	−	−
4	+	−	+	10	+	−	−
5	+	−	+	11	+	−	+
6	+	−	−	12	+	−	+

**Table 4:** Distribution pattern of *C. coli* isolates in pigeons and Turkey.

Bird serial number	Source of samples			Bird serial number	Source of samples		
	Intestinal content	Liver	Skin		Intestinal content	Liver	Skin
(A) Pigeon							
1	+	+	+	9	−	+	−
2	−	−	−	10	+	−	+
3	−	+	−	11	+	−	+
4	−	−	+	12	+	−	+
5	+	−	+	13	+	−	+
6	−	+	−	14	+	−	+
7	−	+	−	15	−	+	−
8	−	+	−	16	−	+	−
(B) Turkey							
1	−	+	+	8	−	−	−
2	−	+	+	9	−	+	−
3	−	+	+	10	+	−	−
4	−	+	+	11	+	+	+
5	−	+	+	12	+	−	+
6	−	+	−	13	−	+	−
7	−	+	+	14	+	+	−

### 3.3. Distribution pattern of *Campylobacter* species isolates in Turkey

*Campylobacter* species were identified in 13% (39/300) of the turkeys, of which 8, 19 and 12% of the positive samples were from the intestinal content, liver, and skin, respectively. *C. jejuni* was detected in the intestinal content (4%), liver (8%), and skin (4%) samples, and the differences between sample types were not statistically significant. Furthermore, *C. coli* was identified in intestinal content (4%), liver (11%), and skin (8%) samples, and the differences between sample types were not statistically significant (Table 2). Sixteen isolates of *C. jejuni* were isolated from 12 turkeys (Tables 1, 2), and different patterns were shown. Three isolates of *C. jejuni* were isolated from the intestinal content, liver, and skin samples of the same turkey (No.2). *C. jejuni* was isolated from both the liver and skin samples from four turkeys (Nos. 4, 5, 11, 12), and separate isolates were isolated from the intestinal content and liver samples of the rest of turkeys (Table 3). Twenty-three isolates of *C. coli* were isolated from 14 turkeys (Tables 1, 2), and different patterns were shown. *C. coli* was isolated from both the liver and skin samples of six turkeys (Nos.1, 2, 3, 4, 5, and 7), *C. coli* was isolated from the intestinal content, liver, and skin samples of the same turkey (No.11) *C. coli* was isolated from the intestinal content and liver samples from turkey No. 14, and separate isolates were isolated from the liver or skin samples of the rest of the turkeys (Table 4).

### 3.4. Occurrence of *Campylobacter* species in poultry shop workers

In humans, *Campylobacter* species were identified in 29% (*C. jejuni* 15%; *C. coli* 14%) of the examined workers using mPCR (Table 1). *Campylobacter* species were detected among workers in percentages of 40, 33.3, and 10% in the 18–30, 31–40, and 41–50 year age groups, respectively, and the differences were significant (*p* = 0.179) (Table 5). *C. jejuni* and *C. coli* were recovered from workers from the 18–30, 31–40, and 41–50 year age groups in percentages of (25%; 15%), (10%; 23.3%), and (6.7%; 3.3), respectively (Table 5).

**Table 5:** Occurrence of *Campylobacter* species in poultry shop workers.

Age (years)	Number of samples	*Campylobacter* species		*Campylobacter jejuni*		*p-value*	*Campylobacter coli*		*p-value*
		No.	%	No.	%		No.	%	
18–30	40	14	35	9	22.5	*p* < 0.05	5	12.5	*p* < 0.05
31–40	30	8	26.7	2	6.7		6	20	
41–50	30	2	6.7	1	3.3		1	50	
Total	100	24	24	12	12		12	12	

## 4. Discussion

Poultry is an important reservoir of food-poisoning microorganisms. In Egypt, the occurrence of *Campylobacter* in poultry farms continues to be a major problem facing poultry production, especially because the poultry industry mainly includes small-scale producers on farms that do not apply biosecurity measures. In the current study, the identification of *Campylobacter* species from the examined samples (*n* = 300) by using the culture method alone or in combination with mPCR was statistically significant. The prevalence rates of *C. jejuni* (26.7%; 31%) and *C. coli* (9.7%; 14.7%) determined by the two methods were statistically significant (Table 1). In contrast, no significant difference was reported between the results obtained with the culture and PCR methods for the detection of *Campylobacter* species in another study (20). The biochemical identification of *C. jejuni* isolated by the culture method mainly depends on the results of the hippurate hydrolysis test, which reacts positively for *C. jejuni* and is negative for other species of *Campylobacter* (21, 22). However, some strains of *C. jejuni* react negatively to the hippurate hydrolysis test as a result of a failure in the transcription of the *hipO* gene (23). On the other hand, sometimes, *C. coli* reacts positively to hippurate hydrolysis as a result of the occurrence of amino acids in the media (24). Hence, the culture method and biochemical reaction are not sufficient to identify *Campylobacter* species, and confirmation by molecular identification is preferred. In contrast to the prevalence of *C. jejuni* recovered from the pigeons in this study (Table 1), lower percentages of *C. jejuni* (11.1 and 8.1%) were obtained in previous studies conducted in California and Croatia, respectively (25, 26). On the other hand, higher percentages (69.1 and 28%) of *C. jejuni* have been reported in other studies conducted in Spain and Italy, respectively (27, 28). Concerning the prevalence of *C. coli* in pigeons, a lower percentage (1.1%) was detected in another study (28).

Compared with previous studies carried out in the United States that have reported the prevalence of *Campylobacter* species (1.6 and 17%) in turkeys (29, 30), our study reported the highest prevalence rate (Table 1). On the other hand, several studies have reported higher percentages (46, and 31.4%) of *Campylobacter* species in Canada and the UK (20, 31), respectively. In this study, a higher prevalence rate of *C. coli* (16%) compared to that of *C. jejuni* (12%) was found in turkeys (Table 1). This result is inconsistent with the results of several studies carried out in Denmark and Hannover (32, 33). In contrast, Noormohamed and Fakhr (29) noted a higher frequency of detection for *C. jejuni* compared with *C. coli*.

In pigeons, significant variations in the occurrence rates of *C. jejuni* and *C. coli* were reported for the intestinal content (15, 4%), liver (4, 13%), and skin (9, 7%) samples (Table 2). In contrast, a previous study showed a higher percentage of positive *C. jejuni* (21.7%) samples recovered from the intestinal content (34). Meanwhile, lower percentages of *C. jejuni* (5.26%) were identified in skin samples in another study (35). In turkey (Table 2), *Campylobacter* species were detected in the liver (19%), skin (12%), and intestinal content (8%). A similar result was reported in another study conducted in Delta Governorates, Egypt (36). However, the occurrence rates of *Campylobacter* species in the liver varied between 9.7 and 30% in previous studies conducted in Germany and Egypt (36, 37). Conversely, higher percentages (55, 26.7%) of *Campylobacter* species were recovered from the skin in previous studies (36, 38). Compared to our findings for the intestinal content (Table 2), higher percentages of *Campylobacter* (16.7%) were reported elsewhere (36) The variation in the occurrence rates of *Campylobacter* species noted in different studies might be attributed to variations in the level of cross-contamination that may occur during the slaughter and evisceration of birds. It is clearly evident that the livers of pigeons and turkeys are potential sources of *Campylobacter* infection, especially when consumed undercooked.

In relation to the rate of *Campylobacter* infections in human population in Egypt, several previous studies documented that Campylobacterios is an important cause of diarrhea in children in the country (39–41). In this respect, up to 85% of children in Egypt were found infected with *Campylobacter* sp. in their first year with annual incidence of 1.2 episodes per year (42–45). Regarding the isolation rate of *Campylobacter* species in Egypt, several studies reported variable percentages (27.55, 5.33, and 18.3%) in Assiut and Aswan Governorates (46–48), respectively. Other studies at various Egyptian governorates reported an isolated rate of 8.5 and 38.09% for *C. jejuni* isolated from occupational workers (42, 49). Moreover, at species level, *C. jejuni* and *C. coli* could be identified in Aswan Governorate at rate of 50% for each (46), while the isolation rate Assiut Governorate (48) was 11.7 and 6.7% for the same species, respectively. Taken into account, in Egypt, farming practices often lack sufficient biosecurity and control which are considering predisposing factors for higher incidence of the pathogen. Among others, it is evident that persons working at poultry shops and dealing with live birds are at high risk of acquiring various zoonotic pathogens, especially during the handling, slaughtering, and evisceration of birds. Therefore, we investigated workers at poultry shops (*n* = 100) to explore the occurrence of *Campylobacter* species among them. Interestingly, the overall occurrence of *Campylobacter* species among the examined workers (Table 5) was 29%. Conversely, several previous studies conducted in in Tanzania, Bangladesh, and Egypt (50–52) have reported lower percentages (9.3, 11.5, and 5.3%) of *Campylobacter*38–40. *C. jejuni* was identified in 15% of the examined workers. Conversely, lower percentages of *C. jejuni* (5.8 and 1.5%) were obtained in other studies conducted in France and India (53, 54). On the other hand, higher percentages of *C. jejuni* (21.4, 63.6%) were obtained in other studies (32, 55). In this study, *C. coli* was recovered from 14% of the workers. Lower percentages of *C. coli* (2.5 and 1.5%) were obtained in other studies (52–54). Higher percentages of *C. coli* (78.5 and 31.8%) were obtained in previous studies (32, 55). The role of asymptomatic persons in the epidemiology of *Campylobacter* is still unclear and further investigation is needed to study the role of carriers in the epidemiology of *Campylobacter*, how long they remain as carriers, and whether nonclinical cases can develop clinical disease. A significant rate of *Campylobacter* infection in relation to the age of the workers was reported in this study. A similar result was obtained in another study conducted in Tanzania (12).

## 5. Conclusion

Based on the results obtained in this study, it is apparent that *Campylobacter* species are circulating in poultry farms in Egypt which might be a risk hazard for humans. Therefore, it is critically important to apply hazard analysis and critical control points at all stages of the production chain until the products reach the consumers. Moreover, the transformation of LBM into chilled poultry markets is recommended. The relatively high occurrence of *Campylobacter* among workers might reflect the poor hygiene practices applied at live poultry shops. Thus, the awareness of poultry shop workers about safe handling practices in the workplace needs to be increased to decrease the possibility of cross-contamination and to prevent the zoonotic transmission of *Campylobacter* infection. Further research, at large scale, is highly recommended for exploring the antimicrobial resistance and genotyping of the circulating strains of *Campylobacter* species from different reservoirs in the Egyptian environment.

## Data availability statement

The original contributions presented in the study are included in the article/Supplementary material, further inquiries can be directed to the corresponding authors.

## Ethics statement

The studies involving human participants were reviewed and approved by Assiut University, Egypt with an ethical approval number of 1717300906. The patients/participants provided their written informed consent to participate in this study. The animal study was reviewed and approved by the Institutional Review Board of the Assiut University (Local ethical approval), Assiut University, Egypt with approval number is 17300906. Written informed consent was obtained from the owners for the participation of their animals in this study.

## Author contributions

AS, AI, MS, EE, and AY designed the idea of the conception, performed the methodology, formal analysis, data curation and supervision besides revision of the manuscript. NA and KA participated in designing of the idea of the conception and drafting of the manuscript. AS, AI, MS, AY, ME-k, and EE drafted the manuscript, prepared the manuscript for publication and revision. All authors have read and agreed to the published version of the manuscript.

## Funding

This study was supported by Princess Nourah Bint Abdulrahman University Researchers Supporting Project number (PNURSP2023R23), Princess Nourah bint Abdulrahman University, Riyadh, Saudi Arabia.

## Conflict of interest

The authors declare that the research was conducted in the absence of any commercial or financial relationships that could be construed as a potential conflict of interest.

## Publisher’s note

All claims expressed in this article are solely those of the authors and do not necessarily represent those of their affiliated organizations, or those of the publisher, the editors and the reviewers. Any product that may be evaluated in this article, or claim that may be made by its manufacturer, is not guaranteed or endorsed by the publisher.
