# Supplementary material for: Circulation of thermophilic Campylobacter in pigeons, turkeys, and humans at live bird markets in Egypt
Source: Front Vet Sci. 2023 May 12;10:1150077. doi: 10.3389/fvets.2023.1150077 (PMC10213357; doi:10.3389/fvets.2023.1150077)
Supplement: Supplementary file 1 [file Data_Sheet_1.docx]

**Supplementary File**

Circulation of thermophilic *Campylobacter* in pigeons, turkeys, and humans at live bird markets in Egypt

Amal S.M. Sayed^1^, Ahmed.I. Ibrahim^2^, Mona M. Sobhy ^3^, Ehab Kotb Elmahallawy *^4^, Noorah Alsowayeh^5^, Khaloud Mohammed Alarjani ^6^, Manal F. El-khadragy ^7^, and Asmaa Gahlan Youseef ^8^

^1^ Department of Zoonoses, Faculty of Veterinary Medicine, Assiut University, Asyut 71515, Egypt.

^2^ Poultry Diseases Department, Faculty of Veterinary Medicine, South Valley University, Qena 83523, Egypt.

^3^ Reproductive Diseases Department, Animal Reproduction Research Institute, El-Haram 12556, Giza, Egypt.

^4^ Department of Zoonoses, Faculty of Veterinary Medicine, Sohag University, Sohag 82524, Egypt.

^5^ Department of Biology, College of Education (Majmaah), Majmaah University, Al-Majmaah, 11952, Saudi Arabia.

^6^ Department of Botany and Microbiology, College of Science, King Saud University, Riyadh 11451, Saudi Arabia.

^7^ Department of biology, College of Science, Princess Nourah bint Abdulrahman University, P.O. Box 84428, Riyadh 11671, Saudi Arabia.

^8^ Zoonoses Department, Faculty of Veterinary Medicine, South Valley University, Qena 83523, Egypt.


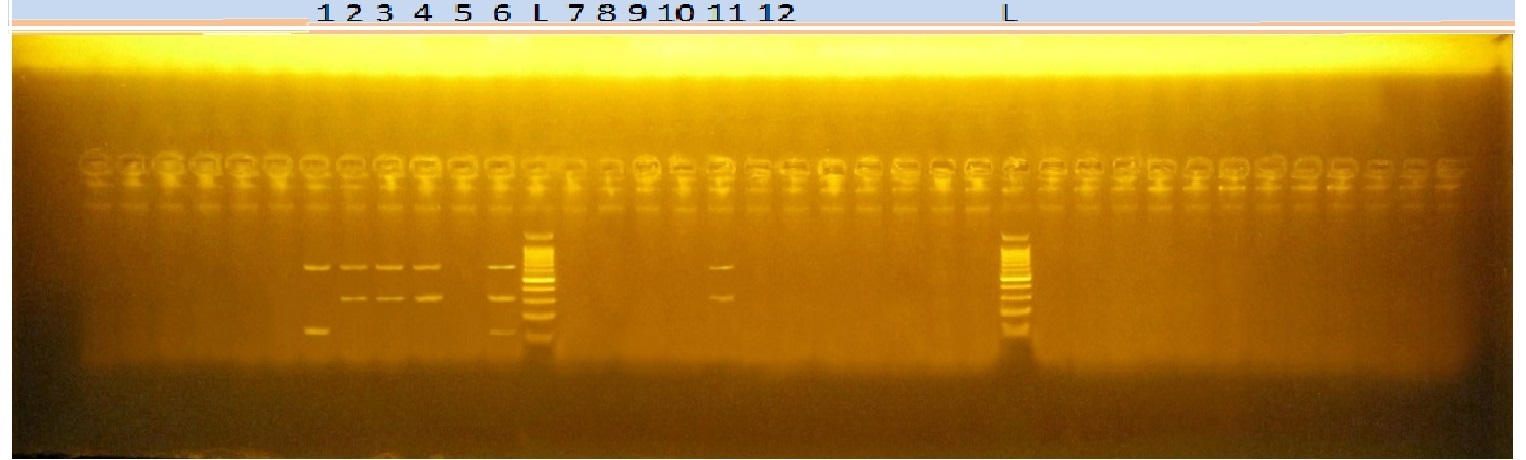
***** Correspondence: * [amalsayed73@aun.edu.eg](mailto:amalsayed73@aun.edu.eg) (A.S.M.S); [eehaa@unileon.es](mailto:eehaa@unileon.es) (E.K.E)

**Supplementary Figure 1.** Agarose gel electrophoresis of *Campylobacter* spp. (650 bp) using *23S rRNA* gene, *Campylobacter jejuni* (323 bp) using *hipO* gene and *Campylobacter* *coli* (126 bp) using *glyA* gene; Lane 1: positive sample for *C.coli*; Lanes 2, 3, 4 and 11: positive samples for *C. jejuni*; Lane 5: Negative control; Lane 6: Positive control of *C.jejuni* and *C.coli* and L: 100bp molecular size marker.
